# The effect of virtual reality glasses used during inhaler treatment on anxiety, fear and vital signs in children: a randomized controlled trial

**DOI:** 10.1007/s00431-026-07077-0

**Published:** 2026-05-19

**Authors:** Yasemin Özyer Güvener, Şeyma Şimşirgil Kara

**Affiliations:** Faculty of Health Sciences, Sinop University, Sinop, Turkey

**Keywords:** Virtual reality glasses, Pediatrics, Inhaler treatment, Anxiety, Fear

## Abstract

This study aimed to determine the effect of virtual reality (VR) glasses used during inhaler treatment on anxiety, fear and vital signs in children. The study had an experimental, randomized controlled design. Participants were assigned to groups using a lottery method based on simple randomization. The random sequence was generated by physically mixing up 30 red (experimental) and 30 white (control) cards. All the data were collected and finalized by two trained researchers (Yasemin Özyer Güvener, a nurse researcher, and Şeyma Şimşirgil Kara, a pediatrician). Participants were recruited using a convenience sampling method from among individuals who met the inclusion criteria. In terms of baseline measurements, the experimental group had significantly higher mean pulse rate, fear scores, and anxiety scores (130.23 ± 14.64, 2.77 ± 1.25, and 4.60 ± 2.04, respectively) compared to the control group (118.83 ± 17.10, 2.07 ± 1.26, and 2.80 ± 2.07, respectively) (*p* = 0.007, *p* = 0.035, and *p* = 0.001, respectively). For post-intervention measurements, the experimental group had significantly lower mean fear and anxiety scores (0.443 ± 0.16 and 0.703 ± 0.22, respectively) compared to the control group (2.39 ± 0.16 and 3.197 ± 0.22, respectively) (*p* < 0.001 and p = 0.001, respectively).

*Conclusion*: It was determined that VR glasses reduced anxiety and fear, increased oxygen saturation, and reduced respiratory and pulse rates. They can thus be used as an alternative method to reduce fear and anxiety levels and balance vital signs during inhalation therapy in children.

**Table:** 

**What is Known:** • *Virtual reality (VR) is an effective non-pharmacological method for reducing pain, anxiety, and fear in pediatric patients during various medical procedures.* • *VR applications create a sense of presence in a virtual world, limiting the impact of anxiety and fear in clinical settings.*
**What is New:** • *Using VR glasses during the administration of inhaled medication significantly reduces anxiety and fear levels in children compared to traditional methods.* • *While evidence for VR use during inhalation therapy was previously limited, this study demonstrates its effectiveness as a promising alternative to improve treatment compliance and physiological responses in pediatric patients.*

**Supplementary Information:**

The online version contains supplementary material available at 10.1007/s00431-026-07077-0.

## Introduction

Children are frequently admitted to health facilities for medical treatment and care [1]. They are most frequently affected by respiratory diseases during the growth period. Inhaler therapy is among the most commonly used methods to treat such diseases. Medicines administered using a mask are frequently used in the treatment of respiratory disorders in children [2].

The use of masks in inhalation therapy is a significant issue. For the treatment to be effective, the mask must be fully placed on the face during use. However, young children may have difficulties wearing masks for various reasons, including the restricted mobility they experience, as well as other anxiety and fear. Children may react negatively to these interventions by crying, becoming restlessness, and refusing treatment [3]. These reactions, however, vary depending on the children’s level of anxiety and distress [4]. Distraction techniques can be used to reduce children’s anxiety when inhaling drugs [5]. However, since inhalation therapy with a face mask is often frightening for children, it can be difficult to provide this treatment effectively and safely. Evidence-based distraction methods effectively ensure efficient inhalation and reduce children's levels of fear. It has been stated that these interventions are effective in reducing anxiety and fear [5].

Virtual reality (VR) glasses are one the methods which can be used to reduce negative emotional behaviors [6]. VR is a tool that uses three-dimensional computer-based technologies that allow an individual to feel as if they are physically present in a virtual environment by altering their sensory experience [7]. The virtual environment developed in virtual reality is provided by head-mounted glasses, consisting of a small screen and a headset [8]. VR is an innovative non-pharmacological tool that can be integrated into pediatric clinical settings to support children during medical procedures. However, its accessibility may vary based on the resources of the healthcare facility. The patient can also be prevented from perceiving the ambient sounds of the hospital by wearing headphones and listened to relaxing music [9].

Studies on the effect of nonpharmacological methods in inhalation therapy, one of the common non-invasive interventions used in children, are limited [10]. VR applications can be employed to reduce anxiety and fear in many areas [7]. This study aimed to determine the effect of using VR glasses during inhaler treatment on anxiety, fear and vital signs in children.

### Study Hypotheses

H_0_: VR glasses have no effect on anxiety, fear, and vital signs in children.

H_1_: VR glasses have an effect on anxiety, fear, and vital signs in children.

## Methods

### Study design

This randomized controlled trial was conducted in the pediatric ward of a state hospital in northern Türkiye. Data were collected between July 2024 and December 2024. Inhalation therapy was administered in the children's rooms. Hertzog stated that 20 participants per group are needed to make the assumptions of homogeneity and normality of variances [11]. Hertzog also assumes that 30 to 40 participants per group are needed to determine the sample size when no significant difference is known. To calculate the estimated sample size in line with the literature, the sample calculation was performed using the G*Power 3.1 package software. With a Type I error of 0.05 and a test power of 0.95 (a:0.05, 1b:0.95), it was decided to include 30 children in each of the experimental and control groups [3]. In this study, the children were divided into two groups: the VR glasses (experimental) group (n = 30) and the control group (n = 30).

### Randomization

**Fig. 1:**
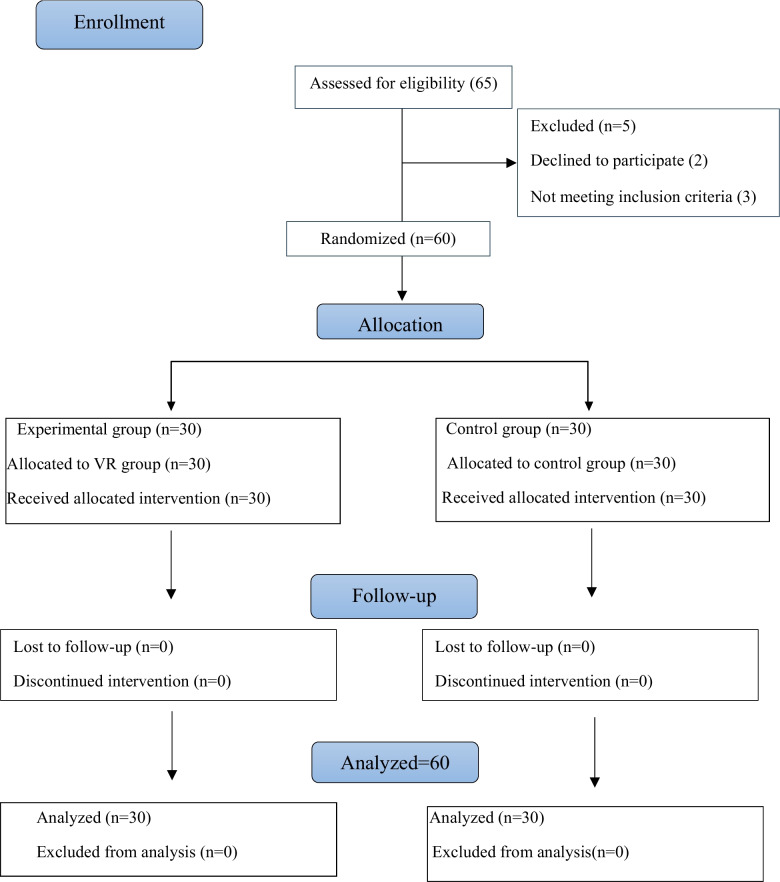
Consort flow diagram of in the study

Participants were assigned to groups using a lottery method based on simple randomization. A random sequence was generated by physically mixing up 30 red (experimental) and 30 white (control) cards. To ensure that how the cards were allotted remained hidden, the cards were placed in an opaque black pouch. The allocation process was managed by an independent assistant who was blinded to the study’s hypothesis. Each card was drawn under supervision, ensuring that group assignment remained unknown to both the researcher and the participant until the moment of allocation. This procedure minimized selection bias. The CONSORT flow diagram is shown in Fig. 1.

### Participants

Children who had to receive inhaler treatment with a mask due to respiratory system disease, who were between the ages of 3 and 5, had an oxygen saturation of 90% or more, and who volunteered to participate, were included in the study. Children with a chronic disease, communication, mental, or neurological disorder, and who had previously received inhaler therapy were not included. The participants were recruited using a convenience sampling method from among individuals who met the inclusion criteria.

### Intervention

After written informed consent was obtained from the parents of the child to receive inhaler medication, the procedures to be performed were explained to the parents. Baseline data, including vital signs (pulse, respiration, SpO₂) and fear and anxiety scores, were collected 5 min before the inhaler treatment. Post-intervention measurements of vital signs, fear, and anxiety were collected immediately after the completion of the inhalation procedure. For the first administration of medication, it was ensured that the child was in a sitting position and that a mask suitable for the face had been selected. The inhalation process took an average of 10 min. To ensure procedural consistency, those administering the treatment underwent standardized training on the study protocol and intervention steps before the commencement of the study. All procedures were carried out following a predefined structured manual to ensure that all participants received the same level of care and that the intervention was applied uniformly. All data were collected by two trained researchers (Yasemin Özyer Güvener, a nurse researcher, and Şeyma Şimşirgil Kara, a pediatrician). Participants were also given no additional concurrent treatments that could affect the outcomes.

#### Virtual reality group

**Fig. 2:**
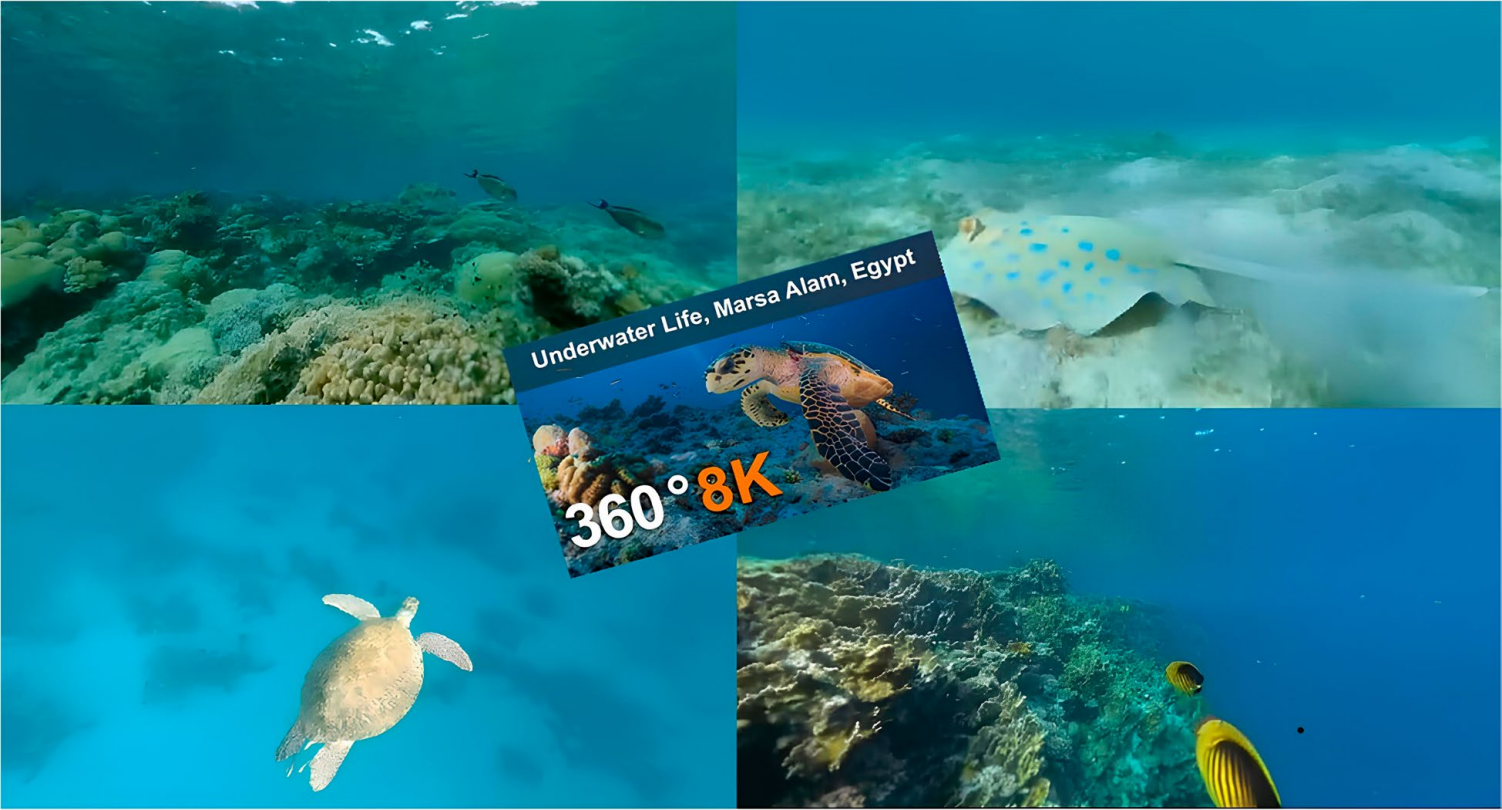
An image of a VR scene shown to the child while receiving inhaler treatment

Before the intervention, the VR glasses were shown to the family, and it was explained how they would be used. The child was able to familiarize themselves with the glasses 2 min before starting the medication. Each child wore the VR glasses for about 10 min while the medication was given through inhalation. Various images, including, for example, images of an “underwater world”, were shown to the participants, accompanied by relaxing music in the background. Figure 2 provides an image of a VR scene shown while the children were receiving inhaler treatment. [Insert Fig. 2 Here].

#### Control group

The children were given inhaled medication with a mask for an average of 10 min, their vital signs (pulse, SPo2, respiration) were recorded, and the Children's Fear Scale (CFS) and Venham's Clinical Anxiety Rating Scale (VCARS) were administered by the researcher before and after the intervention. No intervention was applied to the control group.

### Data collection tools

Data Identification Form: This form consisted of questions on various demographic and clinical characteristics such as age, gender, etc.

**Vital signs form** This recorded parameters such as respiratory rate, pulse rate, and oxygen saturation.

**Venham's clinical anxiety rating scale (VCARS)** This scale was developed by Venham et al. There are eight cards on the scale with two pictures on each card (one of the pictures on each card shows a child with anxiety and the other without anxiety). The cards, numbered from 1 to 8, are shown to the child in order. An anxious picture scores 1 point while a non-anxious picture scores 0 points. The minimum score that can be obtained from the test is 0 and the maximum score is 8. As the score obtained from the test increases, the level of anxiety also increases [12].

Children's Fear Scale (CFS): This scale was developed by McMurty et al. It consists of five facial expressions ranging from a neutral face (0 = “no fear”) to a frightened face (4 = “severe fear”) [13]. The Turkish validity and reliability study of the scale was conducted by Gerçeker et al. (2018) [14].

Before the procedure, the children were given a brief and age-appropriate explanation of the CFS. It was explained that the scale consists of five facial expressions ranging from no fear to the highest level of fear, scored from 0 to 4, where 0 indicates no fear and 4 indicates the highest level of fear. After confirming that the children understood the scale, they were asked to select the face that best represented their level of fear before receiving the inhaler treatment. The question posed to the children was: “Can you choose the face that shows how afraid you are before the inhaler treatment?” The corresponding scores were recorded.

Simultaneously, the parents and the researchers independently rated the child’s pre-procedure fear level through observation using the same CFS facial expressions without seeing the child’s own response, and their scores were recorded.

After the inhaler treatment, the CFS was again administered to the children. This time, they were asked: “Can you choose the face that shows how afraid you were during the inhaler treatment?” Their responses were again recorded using the 0–4 scoring system. In parallel, parents and researchers independently assessed the child’s fear during the procedure, and these scores were also documented.

### Data analysis

Categorical data were summarized as numbers and percentages, and quantitative data were summarized as arithmetic mean and standard deviation. Baseline comparisons of primary outcomes between the control and experimental groups were conducted using an independent samples t-test when the assumption of homogeneity of variances was met, and Welch’s independent samples t-test when this assumption was violated. In addition, Cohen’s *d* effect sizes were calculated and reported. Post-intervention measurements were analyzed using ANCOVA, with baseline (initial) measurements included as a covariate. Estimated marginal means were reported and interpreted. Partial eta squared effect sizes were also calculated and reported. The statistical significance level was accepted as 5% and all statistical analyses were performed using JASP (version 0.18.3, University of Amsterdam).

### Ethical statement

Written permission for this study was obtained from the Human Research Ethics Committee of Sinop University with the decision number 2024/171 dated July 01, 2024. In addition, written permission was obtained from the hospital where the study was conducted. In the study, the parents of the participants were informed about the study, their written consent was obtained, and the participants’ information was kept confidential. The study was carried out in accordance with the Declaration of Helsinki. The study was not registered as a clinical trial. No adverse events or unintended effects were observed during the study.

## Results

The demographic data of the participants are presented in Table 1 and clinic-related data are presented in Table 2.

**Table 1:** Socio-demographic characteristics

	Control	Experimental
	$\overline{\mathrm{X} }$±SD/n (%)	$\overline{\mathrm{X} }$±SD/n (%)
Age of the child	3.43 ± 1.22	3.33 ± 0.99
Mother's age	32.8 ± 5.86	33.27 ± 3.67
Father's age	36.67 ± 5.72	36.17 ± 2.9
Gender		
Female	11 (36.7)	18 (60)
Male	19 (63.3)	12 (40)
Mother's Education Status		
Primary education	3 (10)	3 (10)
High School	13 (43.3)	16 (53.3)
University	14 (46.7)	11 (36.7)
Father's Education Status		
Primary education	4 (13.3)	1 (3.3)
High School	11 (36.7)	13 (43.3)
University	15 (50)	16 (53.3)
Family Structure		
Nuclear Family	29 (96.7)	29 (96.7)
Extended Family	1 (3.3)	1 (3.3)
Other Children in the Family		
Yes	19 (63.3)	16 (53.3)
No	11 (36.7)	14 (46.7)
Economic Status		
Low	2 (6.7)	1 (3.3)
Moderate	18 (60)	26 (86.7)
High	10 (33.3)	3 (10)
Health Insurance		
SSI	24 (80)	29 (96.7)
Paid	4 (13.3)	0 (0)
Other	2 (6.7)	1 (3.3)

$\overline{\mathrm{X} }$: Arithmetic mean, SD: Standard deviation

**Table 2:** Information on health status

	Control	Experimental
	n (%)	n (%)
Diagnosis		
Bronchitis	8 (26.7)	9 (30)
Pneumonia	8 (26.7)	7 (23.3)
Bronchiolitis	10 (33.3)	12 (40)
Other	4 (13.3)	2 (6.7)
Reason for hospitalization		
Shortness of breath	8 (26.7)	11 (36.7)
Cough	11 (36.7)	2 (6.7)
Wheezing	11 (36.6)	17 (56.6)
Previous hospitalization status		
Yes	19 (63.3)	16 (53.3)
No	11 (36.7)	14 (46.7)
Symptoms of respiratory distress		
Shortness of breath	9 (30)	9 (30)
Cough	5 (16.7)	2 (6.7)
Tachypnea	2 (6.7)	6 (20)
Wheezing	14 (46.7)	13 (43.3)

The findings related to group comparisons are presented in Table 3. In terms of baseline measurements, the experimental group had significantly higher mean values for pulse rate, fear score, and anxiety score (130.23 ± 14.64, 2.77 ± 1.25, and 4.60 ± 2.04, respectively) compared to the control group (118.83 ± 17.10, 2.07 ± 1.26, and 2.80 ± 2.07, respectively) (*p* = 0.007, *p* = 0.035, and *p* = 0.001, respectively; Table 3).

**Table 3:** Group comparisons in terms of baseline measurements

	Control	Experimental			
	$\overline{\mathrm{X} }$±SD	$\overline{\mathrm{X} }$±SD	t	p^a^	Cohen d
Pulse	118.83 ± 17.1	130.23 ± 14.64	−2.774	0.007	−0.716
Respiration	30.8 ± 6.7	32.57 ± 4.59	−1.191	*0.238	−0.308
SpO2	93.87 ± 3.06	94.73 ± 2.46	−1.209	0.232	−0.312
Fear score	2.07 ± 1.26	2.77 ± 1.25	−2.162	0.035	−0.558
Anxiety score	2.8 ± 2.07	4.6 ± 2.04	−3.385	0.001	−0.874

X̄: Mean; SD: Standard deviation; a: Independent samples t-test; *: Welch’s independent samples t-test

Post-intervention measurements were analyzed using ANCOVA, with baseline (initial) measurements included as a covariate, and the estimated marginal means are presented in Table 4. According to the ANCOVA results, the differences between groups in terms of fear and anxiety scores were statistically significant. The post-intervention mean scores for fear and anxiety in the experimental group (0.443 ± 0.16 and 0.703 ± 0.22, respectively) were significantly lower than those in the control group (2.39 ± 0.16 and 3.197 ± 0.22, respectively) (*p* < 0.001 and *p* = 0.001, respectively; Table 4).

**Table 4:** Group comparisons for post-intervention measurements

	Control	Experimental			
	$\overline{\mathrm{X} }$±SD	$\overline{\mathrm{X} }$±SD	F	P^*^	Partial Eta Squared
Pulse	125.325^a^ ± 1.99	125.075^a^ ± 1.99	0.007	0.931	< 0.001
Respiration	30.242^a^ ± 0.57	28.791^a^ ± 0.57	3.180	0.080	0.053
SpO2	96.328^a^ ± 0.25	97.005^a^ ± 0.25	3.545	0.065	0.059
Fear score	2.390^a^ ± 0.16	0.443^a^ ± 0.16	75.912	< 0.001	0.571
Anxiety score	3.197^a^ ± 0.22	0.703^a^ ± 0.22	60.695	< 0.001	0.516

X̄: Mean; SD: Standard deviation; a: Estimated marginal mean adjusted for covariate(s)

ANCOVA analysis with baseline measurements included as covariate(s)

## Discussion

The present study determined that using VR glasses during the administration of inhaled medication reduced anxiety and fear in the experimental group compared to the control group, had positive results on vital signs. VR applications, which are among the visual distraction methods, limit the number of stimuli from the real environment while increasing stimuli from a virtual environment, thus increasing the sense of being present in this virtual world [15, 16]. In addition, recent systematic reviews and meta-analyses have demonstrated that VR is an effective non-pharmacological method for reducing pain, anxiety, and distress in pediatric patients across various clinical settings [16–18].

In the present study, the mean pulse rate after inhalation therapy was found to be lower in the experimental group compared to the control group. Similarly, pulse rate has varied in other studies [10, 19]. Recent studies have also reported that VR interventions are associated with decreased heart rate and improved physiological responses in children undergoing medical procedures [20, 21].

In our study, the respiratory rate changed and decreased more in the experimental group than in the control group. A previous study found that respiratory rate decreased during inhalation therapy [10]. The present study determined that the oxygen saturation rate was higher in the experimental group compared to the control group. Another study reported that the mean oxygen saturation was the same throughout the entire inhalation treatment [10]. Similarly, more recent studies have indicated that VR may positively influence physiological parameters, including oxygen saturation, by reducing stress responses and improving treatment compliance. The findings in the literature support those of the present study [22, 23].

The present study found that the fear scores of the children in the experimental group were lower after inhalation treatment compared to the control group. In similar studies where different techniques were used to distract attention, it was reported that fear scores decreased [5, 10, 19]. Using VR glasses during vaccine administration has been reported to be effective in reducing fear [9]. In line with these findings, recent studies using VR have consistently demonstrated significant reductions in fear during various pediatric procedures such as injections and venipuncture [16, 24, 25]. The findings in the literature support those of the present study.

In the present study, the anxiety scores in the experimental group decreased more than in the control group. A previous study using distraction techniques also reported a decrease in anxiety scores [25, 26]. In a study in which VR glasses were used in anxiety and behavior management in children undergoing dental treatment, it was reported that the anxiety score decreased [27]. Another study using VR glasses also reported that anxiety levels decreased [22]. Supporting these findings, recent systematic reviews and meta-analyses have confirmed that VR is effective in reducing anxiety levels and improving behavioral cooperation in pediatric patients [20, 21]. The findings in the literature support those of the present study.

Although the number of studies on the use of VR in children has increased in recent years [28], evidence specifically focusing on VR use during inhalation therapy remains limited [29]. Considering that situations such as crying and not keeping the mask on the face due to fear and anxiety may negatively affect the treatment process, it is promising that the alternative method used in this study reduced anxiety and fear and had a positive effect on treatment.

### Limitations

This study has some limitations. Since the study was conducted with children aged 3–5 years, the findings are limited to this age group. In this study, the fear and anxiety scores were evaluated by the researchers, and the lack of children's self-assessment is a limitation of the study. In addition, in the study, children only experienced VR glasses once, so the results were limited to this experience.

Another limitation of this study was the statistically significant difference in baseline pulse rate and psychological scores between the groups despite randomization. However, this imbalance was statistically addressed by using ANCOVA to ensure the reliability of the intervention's effects.

A further limitation is the lack of blinding of the researchers who collected the outcome data (fear and anxiety scores). Since the assessors were aware of group assignments during data collection, this may have introduced detection bias. Although standardized and validated scales were used to reduce subjectivity, the absence of assessor blinding remains a potential source of bias.

## Conclusion

The present study found that the use of VR glasses effectively reduced children's fear and anxiety levels during inhalation therapy, regulated vital signs, and positively affected the duration of inhaler administration. This study has important implications for pediatrics. This technique can be used as an alternative method to reduce fear and anxiety levels during inhalation therapy in children. The use of such interventions should be considered in order to enhance the patient's compliance with the procedure and reduce the negative effects of anxiety and fear experienced by children during the treatment process.

## Supplementary Information

Below is the link to the electronic supplementary material.

ESM 1 (PDF 2.01 MB)

ESM 2 (DOC 219 KB)

## Data Availability

The datasets used and/or analysed during the current study are available from the corresponding author on reasonable request.
