# Supplementary material for: The effect of virtual reality glasses used during inhaler treatment on anxiety, fear and vital signs in children: a randomized controlled trial
Source: Eur J Pediatr. 2026 May 19;185(6):410. doi: 10.1007/s00431-026-07077-0 (PMC13183728; doi:10.1007/s00431-026-07077-0)
Supplement: Supplementary file 1 — (PDF 2.01 MB) [file 431_2026_7077_MOESM1_ESM.pdf]

POLEN TERCÜME
Akademik Tercüme & Proof Reading
aynı dili konuşmak için

CERTIFICATE OF NATIVE SPEAKER PROOFREADING

18 April 2026
DATE

Dear Editor

THE MANUSCRIPT: **"THE EFFECT OF VIRTUAL REALITY GLASSES IMPLEMENTATION DURING INHALER TREATMENT ON ANXIETY, FEAR AND VITAL SIGNS IN CHILDREN: A RANDOMIZED CONTROLLED TRIAL"** HAS BEEN PROOFREAD BY OUR EXPERIENCED NATIVE SPEAKER PROOFREADER DAVID DUFFY.

POLEN TRANSLATION CO.
ACADEMIC TRANSLATION AND PROOFREADING
DR. AYZAT MADİKOVA ÖZER

Adress: Fulya Mah. Büyükdere Cad. No: 74 /D
Torun Center Kat: 3 Ofis :10 Şişli / İstanbul
Mecidiyeköy V.D. V.No: 27464672222
